# Ten Simple Rules for a Community Computational Challenge

**DOI:** 10.1371/journal.pcbi.1004150

**Published:** 2015-04-23

**Authors:** Iddo Friedberg, Mark N. Wass, Sean D. Mooney, Predrag Radivojac

**Affiliations:** 1 Department of Microbiology, Miami University, Oxford, Ohio, United States of America; 2 Department of Computer Science and Software Engineering, Miami University, Oxford, Ohio, United States of America; 3 Centre for Molecular Processing, School of Biosciences, University of Kent, Canterbury, Kent, United Kingdom; 4 The Buck Institute for Research on Aging, Novato, California, United States of America; 5 Department of Computer Science and Informatics, Indiana University, Bloomington, Indiana, United States of America

In science, the relationship between methods and discovery is symbiotic. As we discover more, we are able to construct more precise and sensitive tools and methods that enable further discovery. With better lens crafting came microscopes, and with them the discovery of living cells. In the last 40 years, advances in molecular biology, statistics, and computer science have ushered in the field of bioinformatics and the genomic era.

Computational scientists enjoy developing new methods, and the community encourages them to do so. Indeed, the editorial guidelines for *PLOS Computational Biology* require manuscripts to apply novel methods. However, it is often confusing to know which method to choose: which method is best? And, in this context, what does “best” mean?

To help choose an appropriate method for a particular task, scientists often form community-based challenges for the unbiased evaluation of methods in a given field. These challenges help evaluate existing and novel methods, while helping to coalesce a community and leading to new ideas and collaborations.

In computational biology, the first of these challenges was arguably the Critical Assessment of protein Structure Prediction, or CASP [1], whose goal is to evaluate methods for predicting three-dimensional protein structure from amino acid sequence. The first CASP meeting was held in December of 1994, following a “prediction period” where members of the community were presented with protein amino acid sequences and asked to predict their three dimensional structures. The sequences that were chosen had recently been solved by X-ray crystallography but had not been not published or released until after the predictions from the community were made. Since the first CASP, we have seen many successful challenges, including Critical Assessment of Function Annotation (CAFA) for protein function prediction [2], Critical Assessment of Genome Interpretation (CAGI) (for genome interpretation) [3], Critical Assessment of Massive (originally “Microarray”) Data Analysis (CAMDA) (for large-scale biological data) [4], BioCreative (for biomedical text mining) [5], the Assemblathon (for sequence assembly), and the NCI-DREAM Challenges (for various biomedical challenges), amongst others [6].

Computational challenges also help solve new problems. While the original CASP experiment was developed to evaluate existing methods applied to current problems, other communities often look at other areas for which there are no existing tools. These challenges have spread successfully to industry, and companies such as Innocentive [7] and X-Prize [8] offer large prizes for solving novel questions.

Because these challenges are, on one hand, an exercise in community collaboration, and on the other, a competition, organizing a challenge is littered with difficulties and pitfalls. Having served as organizers, predictors, and assessors within several existing communities, we present ten rules we believe should be observed when organizing a computational methods challenge:

## Rule 1: Start with an Interesting Problem and a Motivated Community

Organizers of community challenges should start with an active community studying an important, non-trivial problem, and a good number of published tools that solve this or a similar problem using different approaches. Ideally, the challenge should be based on real data and the problem itself should be compelling. It is best to organize the community challenge around a meeting, whether adding the challenge on to an already scheduled event, or establishing a meeting especially for the challenge. Advertising a challenge without having the people to build on may doom your effort before it starts. Ensure you have a critical mass of predictors who are interested before you decide to move forward.

## Rule 2: Make Sure You Have Organizers, Data Providers, and Assessors Available before You Begin

The logistics of a challenge should be handled by separate entities, ideally comprising different people, to minimize potential conflicts of interest. These entities are the *data providers*, who give the testing data on which the methods are to be tested; the *assessors*, who assess the performance of the methods; the *organizers*, who provide the logistic infrastructure for the challenge, and the *predictors* are those who perform the predictions and whose predictions are assessed. Finally, there is a *steering committee*, composed of members who are knowledgeable in the field, but have no stake in the challenge. The members of the steering committee should offer different perspectives to the organizers on everything from rules to logistics, and thus help to better guide the challenge. The organizers should hold regular meetings with the steering committee, report progress, and identify possible faults along the way, and the roles should be sufficiently separated to ensure integrity. Everyone involved should be aware that there is a lot of work to be done over an extended period of time, and that during “crunch periods” such as challenge assessments, the work can take 100% of the time of several people over a few weeks. Even during calmer times, prepare for an extra workload that includes advertising, organizing, writing rules, developing metrics, and developing supporting software and web sites.

## Rule 3: Develop Reasonable Rules, but Be Flexible in Their Application, Especially the First Time

Work with your community and steering committee to come up with reasonable rules for the challenge, but understand that to recognize scientific impact, unforeseen changes will be required, particularly during the first iteration. These rules should be jointly developed by the organizers and the assessors and should be shared with the community for feedback. Learn from the first challenge, and change as necessary in future iterations to adopt rules that fit the questions at hand and the community's ability to address those questions.

## Rule 4: Carefully Consider Your Assessment Metrics

Good, unbiased assessment of the methods is critical to ensuring a successful challenge. Assessors should develop and publish their metrics early, and community input should be collected and used to refine them. The software that the assessors will use to evaluate predictions should be freely available. If possible, recruit assessors who are known and respected by your community. For some challenges, assessment is obvious, and only a single metric is needed. For others, the assessment methods can be more subjective and therefore contentious. If appropriate to your challenge, develop several complementary assessment methods. Take care to keep your assessment metrics easily interpretable. Metrics that are too complex and hard to explain can defeat the purpose of a community challenge.

## Rule 5: Have a Publication Plan

Try to have a publication plan prior to the challenge. Having the backing of a journal willing to publish papers from your challenge may help draw more people to the challenge. Of course, you should ensure that any manuscript is properly peer-reviewed. In CAFA, we availed ourselves of the special supplement mechanism provided by some journals [9]. Typically the papers are a publication of some of the participating methods, and those are authored by the method developers. A *flagship paper*, which provides a broader view of the challenge and participating methods, and authored by all participants, should also be included. You may use several publishers and different journals.

## Rule 6: Encourage Novelty and Risk-Taking

When creating a challenge, and especially with an ongoing challenge, predictors may gravitate toward marginally improving upon what worked in the past, rather than taking risky innovations. It is up to the organizers to encourage risk-taking, as that is where innovations usually originate. The challenges should have some novel edge to them to encourage the concurrent development of significantly novel methods. Also, the time given between challenges should be long enough to allow for the development of new methods, as well as substantial improvements to existing methods. (Typically 2–3 years between major challenge events). Finally, avoid “penalizing” methods that are not as competitive. This can be done by allowing authors to withdraw from the challenge or opt not to have their results published, thus allowing them to improve their methods and avoid a penalty from having a poor score publicly associated with their method.

## Rule 7: Build, Maintain, and Expand Your Community

Holding regular meetings based on your challenge builds community, encourages collaborations, and generally helps your effort become sustainable. Note that organizing meetings can seriously impact your time. Therefore, make sure that you and your collaborators are up for the effort. For more information, see *Ten Simple Rules for Organizing a Scientific Meeting* [10]. Have a meaningful and well-maintained website as a go-to resource for members of your community. Advertise your effort by presenting it at conferences, and over social media. Finally, seek feedback from your community after each event. Seeking feedback will help you understand what you are doing well, and what you may be able to improve. To facilitate a large number of honest responses an anonymous survey is the best way to gain feedback: use tools such as SurveyMonkey or SurveyGizmo. Also, do not allow your challenge to become stale. It may be exciting to gradually but constantly innovate by digging deeper and addressing more challenging aspects of your core problem.

## Rule 8: Seek Funding

Conferences and the challenge effort itself will need funding to sustain growth and existence. You should treat the challenge just like any other research project and seek sustainable funding for it. Work to convince funding agencies and commercial supporters that your challenge is timely and important and could make tangible contributions. Letters of support from challenge participants are crucial here. If possible, you should turn your results into new science where you and your community will test new emergent hypotheses. To make the challenge more transparent and help in the scientific research related to your challenge, you should provide assessment and other relevant software the community can use. Also, urge your participants to release their software to the public. Having tangible, useful products resulting from your challenge will serve the community, as well as help attract funding.

## Rule 9: Give Scientific Credit to the Predictors

One temptation of organizers of conferences, community challenges, or community challenge manuscripts is to somehow give the organizers scientific credit for the work of the data providers and predictors. Predictors and data providers should be celebrated as authors on manuscripts, speakers, and future organizers. Creating an environment where organizers (and not participants) are celebrated will only result in less impactful results, lower participation, and the overall quality of the ongoing challenge may be weakened. At the same time it is important to avoid ranking labs; instead, rank approaches: in the end, it is the competitive drive that motivates many of the predictors in these experiments. While this is well understood by both the community and organizers, calling the challenge and/or treating it like a personal competition will have the likely outcome of stifling risk.

## Rule 10: Prepare for an Incredible Ride, and Have Fun

If your effort is successful, you will be looked to by a community made up of dozens of groups, each group seeking to establish that their method is successful in the challenge. Naturally, in such a competitive environment, tensions and disagreements will arise—over the rules, the metrics, the challenge data, and anything that can be changed. Be patient and understanding, and most importantly, be attentive to criticisms and to the possibility of change. At the same time, after establishing rules, data, and assessment metrics, stick to your guns for the duration of the challenge round, unless you discover a major mistake that could render the challenge meaningless or grossly unfair. You can always fix lesser problems in the next round. You cannot, and therefore should not, aim to please everybody. Be patient and remember that the predictors are working hard and have a lot at stake, which may frustrate them when the assessment is not all they expected. Always remember that the challenge you are organizing is intended to improve methods through a friendly competition, and that you are involved in a community that, when all is said and done, should be a collegial one. Remember to have fun!

## Conclusions

Overall, we believe that if you follow these guidelines, you will be well on your way to helping improve tools and methods through community driven challenges. Make the scientific goals of the challenge abundantly clear and do not try to game the system to profit from the challenge itself. It is hard work, and may initially be unrewarding. The end result, however, can be as rewarding as any in science.
